# Role of thymic stromal lymphopoietin in the pathogenesis of lumbar disc degeneration

**DOI:** 10.1097/MD.0000000000007516

**Published:** 2017-07-28

**Authors:** Yu Wang, Xiao-Dong Yi, Chun-De Li

**Affiliations:** Department of Orthopaedics, Peking University First Hospital, Beijing , P.R. China.

**Keywords:** Aggrecan, lumbar disc degeneration, nucleus pulposus cells, thymic stromal lymphopoietin, Type II collagen

## Abstract

**Background:**

This study aims to investigate the role of thymic stromal lymphopoietin (TSLP) in the pathogenesis of lumbar disc degeneration (LDD).

**Methods:**

Nucleus pulposus tissues were collected from 77 LDD patients (the case group), in addition, normal tissues were extracted from 21 patients suffering from lumbar fractures (the control group). Immunohistochemistry was applied in order to detect TSLP positive expression. In accordance with varying transfection, the cells were divided into TSLP-siRNA, TSLP-siRNA + TSLPR-siRNA, control, blank, anti-TSLPR, and IgG groups. Western blotting was used in order to detect TSLP expression in tissues, and TSLP and type II collagen (COL2AL) in cell culture media were detected using enzyme linked immunosorbent assay (ELISA). Cell viability was measured using a MTT assay. Aggrecan levels were detected using antonopulos, and cell apoptosis was determined using flow cytometry.

**Results:**

TSLP-positive expression was found to be significantly higher in the case group compared with the control group. LDD patients’ Pfirrmann grades and preoperative visual analogue scale (VAS) scores were associated with TSLP-positive rate. Cells transfected with TSLP-siRNA and TSLPR-siRNA plasmids exhibited lower TSLP and thymic stromal lymphopoietin receptor (TSLPR) protein expression compared with the control and blank groups. Compared with the control and blank groups, there was significantly higher cell viability, lower cell apoptosis, and higher COL2AL and Aggrecan levels in the TSLP-siRNA, anti-TSLPR, and TSLP-siRNA+TSLPR-siRNA groups; there were significant differences between the TSLP-siRNA, anti-TSLPR, and TSLP-siRNA+TSLPR-siRNA groups and IgG group (all *P* < .05)

**Conclusion:**

Our study provides evidence for the hypothesis that TSLP could reflect the histological severity of LDD, and TSLP-siRNA and, TSLPR-siRNA could inhibit apoptosis of nucleus pulposus cells. The evident information obtained from the investigation could lead the way for new therapeutic approaches regarding LDD treatment.

## 1 Introduction

Lumbar disc degeneration (LDD), the main cause of lumbago and backache, has a serious impact on general public health.^[1]^ A pathological study on LDD showed that the main causes of LDD could be traced back to genetic factors in addition to the external factors such as smoking, excessive spinal mechanical load, and intervertebral disc damage.^[2]^ The current treatments to alleviate the LDD pain include nonsurgical therapy and surgical therapy.^[3]^ Bone marrow mesenchymal stem cells (BMSCs) transplantation and axial dispersion treatment applied have been reported to have an apparent reversal effect on LDD in animal experiments.^[4]^ At present, a significant correlation between immune responses and the development and progression of LDD has been discovered. This has raised considerable concerns like sciatica, paraesthesia, numbness, or weakness in LDD treatment.^[5]^ Thymic stromal lymphopoietin (TSLP), utilization as a cell growth factor, was initially discovered in mouse thymic stromal cell line (MTSC). ^[6]^

TSLP, a 4-helix bundle structural protein, is similar in structure with interleukin-7 (IL-7). There are many similarities between TSLP and IL-7 in terms of features, but TSLP can stimulate lymphocyte proliferation without relying on IL-7.^[7,8]^ It has been widely reported that TSLP can aid in activating the antigen recognition molecules such as dendritic cells (DC cells) that aid in transforming T cells into T Helper Type 2 cells (Th2-type).^[6]^ There have been numerous studies over the recent years, which primarily focused on exploring the association between TSLP and allergic inflammatory diseases such as asthma, allergic skin, and mucous membrane disease.^[6,9]^ However, TSLP activation in DC cells and the differentiation of Treg cells had a significant impact on the development and progression of autoimmune diseases.^[10]^ As LDD is associated with inflammatory diseases,^[11]^ this study intends to explore the specific role and mechanism of TSLP in LDD so as to discover the effective targets in LDD treatment, which could further improve the chance of successful prevention and diagnosis of LDD.

## 2 Materials and methods

### 2.1 Ethics statement

The study was performed with consent from all participating patients. This study was conducted in accordance with the relative ethical standards and approvals from the Ethics Committee of Peking University First Hospital, following the standard of The National Research Council.

### 2.2 General information

Between June 2013 and January 2015, nucleus pulposus tissues of 77 LDD patients (39 men and 38 women; mean age: 41.69 ± 8.15 years) who were hospitalized in Peking University First Hospital were elected for the case group. Among them, Spengler typing was used for categorizing the patients into 25 cases of protrusion, 29 cases of rupture type, and 23 cases of free type.^[12]^ According to the Pfirrmann grade system,^[13]^ patients were divided into type I (n = 14), type II (n = 20), type III (n = 21), and type IV (n = 22). In adherence with a 100 mm visual analogue scale (VAS) for testing sciatic nerve pain intensity,^[14]^ the average preoperative VAS score was 6.30 ± 1.88. This could be further divided into mild (≤3.4), moderate (3.4–7.5), and severe (≥7.5).^[15]^ The inclusion criteria were as follows: patients without long-term manual labour history; patients without any history of spinal injury; patients who didn’t experience constant backache; patients without lumbago and spine malformation. Exclusion criteria: patients with tumor and autoimmune diseases, chronic infectious diseases, and patients with leg pain but without osphyalgia.^[16]^

Additionally, nucleus pulposus tissues of 21 lumbar vertebral bone fracture patients (13 men and 8 women; mean age: 38.67 ± 7.12 years) were extracted as controls. These patients did not experience any back and leg pain. Patients in the control group had no clinical manifestations and symptoms of LDD as well as no significant degenerative changes in nucleus pulposus tissues post magnetic resonance imaging (MRI) examination. There was no statistical difference observed in terms of age and sex, between the case group and the control group (both *P* > .05).

### 2.3 Immunohistochemistry

Nucleus pulposus tissues were fixed with a 4% paraformaldehyde solution, followed by deparaffinization of paraffin sections. Antigen was repaired under high pressure and at high temperature conditions. The tissues were sealed with normal goat serum. Rabbit polyclonal antibody-TSLP antibody (1:500, ab47943, Abcam Inc., Cambridge, MA) was added and fully mixed with 10 μL of biotin goat anti-rabbit secondary antibody (BA1003, 1:200, BOSTER Biological Technology Co., Ltd., Hubei, China).

Following this, the mixture was used to process the sections over a period of 30 minutes, and then underwent 4 °C overnight inoculation. For developing color diaminobenzidine (DAB) chromogenic (Beijing Cellchip Biotechnology Co., Ltd., Beijing, China) was used. In regards to mouse IgG as blank control, sections were observed under an optical microscope. The cytoplasm was stained using TSLP. Yellow-like particles were regarded as TSLP-positive. From each section, 5 high-power fields (×400) were chosen, and 100 cells were counted in each field. The percentage of TSLP-positive cells/total cells >10% was regarded as positive, while <10% was negative.^[17]^ Two individuals independently estimated the immunohistochemistry results using the double blind method.

### 2.4 Western blotting

The tissue protein from both the case group and the control group were extracted by using the conventional splitting method, and the concentration was testified using a bicinchoninicacid (BCA) kit. 1× Sodium dodecyl sulfate-polyacrylamide gel electrophoresis (SDS-PAGE) loading buffer was added to tissue and the protein were boiled to obtain protein degeneration. SDS-PAGE gel electrophoresis was performed in order to obtain equivalent protein samples. Next, the protein was transferred onto a polyvinylidenefluoride (PVDF) membrane and sealed with bovine serum albumin (50 g/L) for 1 hour. Subsequently, TSLP antibody (at a ratio of 1:200, ab188766, Abcam Inc., Cambridge, MA) and thymic stromal lymphopoietin receptor (TSLPR) antibody (at a ratio of 1:200, ab153480, Abcam Inc., Cambridge, MA) were added to culture the protein at 4 °C overnight. The proteins were washed with TBST buffer solution and cultured with HRP-labeled secondary antibody (at a ratio of 1:500, ab6734, Abcam Inc., Cambridge, MA) for 1 hour. Next, the proteins were washed with TBST another time. Finally, the protein was developed with electro-chemic luminescence (ECL) and photographed under a microscope. Consequently, the above-mentioned method was applied to verify the effectiveness of silencing TSLPR and TSLP.

### 2.5 SiRNA design and quantitative real-time polymerase chain reaction (qRT-PCR)

TSLPR siRNA and TSLP siRNA were coconstructed by the laboratory and Shanghai Genechem Genetic Biological Technology Co., Ltd (Shanghai, China). TSLPR-specific target sequence was used to design 3 different siRNAs in order to avoid an off-target effect. Similarly, 3 different types of siRNAs were designed for TSLPR as well as for negative control. In order to enhance siRNA stability, a dTdT-suspended end was also designed. For RNA interference the above mentioned sequences were connected with lentivirus vectors pGLVU6/GFP. The bacteria transformed and bacterial plasmids were extracted in large quantities. Two hundred and fifty microliter of serum-free Opti-MEM was used to dilute equivalent amount of corresponding siRNA or negative control (Thermo Fisher Scientific Inc. Waltham, MA). The siRNA and negative controls were then introduced in cells to increase the plasmid concentration to 50 nM, which was cultured at room temperature for 5 minutes. This was followed by dilution with 250 μL of serum-free Opti-MEM and mixing with, a cultured 5 μL amount of Lipofectamine 2000 at room temperature for 5 minutes Opti-MEM and Lipofectamine 2000 then fully mixed. The mixture was further cultured for another 20 minutes and introduced into culture wells containing cells. After culturing the cells for 6 to 8 hours, the culture medium was replaced with a completely new medium to continue the cell culturing process. After a day, the total RNA underwent reverse transcription according to the specification of miScript II RT Kit (QIAGEN, Hilden, Germany). The qRT-PCR system was also in accordance with specification of a kit (Thermo Fisher Scientific Inc. Waltham, MA) as well as the primer sequence was shown in Table 1. The reaction system was as follows: predegeneration at 95 °C for 30 seconds, degeneration at 95 °C for 10 seconds, annealing at 60 °C for 20 seconds, extension at 70 °C for 10 seconds, 40 cycles. The qRT-PCR platform (Bio-Rad iQ5; Bio-Rad Laboratories, Inc., Hercules, CA) was used. With β-actin as internal reference, using a relative quantitative method the relative expressions of target genes were shown as 2^−ΔCt^. After which, the percentages of expressions related to *TSLPR* and *TSLP* genes in β-actin were analyzed in order to identify how much *TSLPR* and *TSLP* gene were silenced by siRNA. Each experiment was repeated 3 times.

**Table 1:**
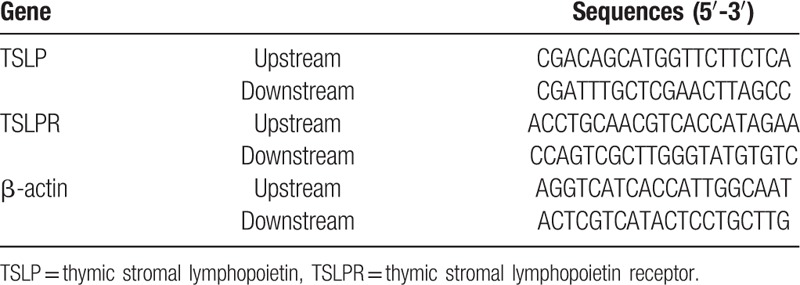
Primer sequences for quantitative real-time polymerase chain reaction.

### 2.6 Isolated culture and grouping of human nucleus pulposus cells

The nucleus pulposus tissues of the LDD patients were extracted and cut into small pieces (0.5 mm in length) in aseptic conditions. The tissue pieces were rinsed with Hams F12 + 10% FBS (Gibco Company, Grand Island, NY) 3 times, and were accordingly inoculated in a culture flask with a basal area of 20 cm^2^ for culturing at 37 °C. After the cells had been isolated and cultured for a week, the cells were passaged and cultured if the cell fusion reached up to 80%. The nucleus pulposus cells were accordingly divided into the TSLPR-siRNA + TSLP-siRNA groups (cells transfected with TSLPR siRNA1 and TSLP-siRNA2), the TSLP-siRNA group (cells transfected with TSLP-siRNA2), the control group (cells transfected with negative control siRNA), and the blank group (cells received no treatment). Two hundred fifty microliter of serum-free Opti-MEM (Gibco Company) diluted equivalent corresponding with siRNA plasmid or negative control siRNA, was then introduced to raise the plasmid concentration up to 50 nM. After culturing at room temperature for 5 minutes the process was followed by dilution with 250 μL of serum-free Opti-MEM and mixing with 5 μL of Lipofectamine 2000, culturing at room temperature for 5 minutes. At the point when Opti-MEM and Lipofectamine 2000 were fully mixed, the mixture was then further cultured for 20 minutes and added to culture wells containing cells in them. After culturing the cells for 6 to 8 hours, the culture medium was replaced with completely new medium to continue cell culturing. At the same time, the nucleus pulposus cells that were not transfected by any plasmids and were still in good condition were inoculated into 6-well plates for culturing for 24 hours. Followed by the addition of the cells to anti-TSLPR (5 μg/m) or homologous and unrelated IgG antibody (R&D Systems, Inc. Minneapolis, MN, USA), for diving them into the anti-TSLPR group and the IgG group.

### 2.7 Immunofluorescence staining

Once the nucleus pulposus were cultured with TSLP for 48 hours, the cells were then cultured on a sterile cover slip. Immunofluorescence staining was employed in order to detect the expression of TSLP after transfection. Once the cells multiplied and fused, the cover slip was fixed by immersing in 4% paraformaldehyde at room temperature for 10 minutes, and followed by sealing with block buffer for 45 minutes. Subsequently, TSLP-monoclonal antibody was added to cells (at a ratio of 1:400, ab115700, Abcam Inc., Cambridge, MA) to be diluted and cultured at 4 °C overnight. Fluorescein-labeled CY-3 goat anti-rabbit secondary antibody (1:500, ab10812, Abcam Inc., Cambridge, MA) was added in order to dilute the cells and culture the solution at room temperature in the dark. After observation under a fluorescence microscope, cells exhibiting red fluorescence were deemed as TSLP-positive and the TSLP-positive rate was calculated.

### 2.8 Enzyme linked immunosorbent assay (ELISA)

The culture medium was collected 48 hours after nucleus pulposus cells were transfected with TSLPR siRNA. The concentrations of TSLP and COL2AL in serum samples were testified according to the specification of ELISA kit (PeproTech Company, Rocky Hill, NJ). The operation was conducted as follows: the antibody was diluted to the concentration of 1 μg/mL using the ELISA coating buffer, and subsequently added into 96-well plates at 4 °C overnight (concentration of 100 μL/well). The coating buffer was removed and the plates were washed 3 times. A total of 150 μL of blocking solution was added in each well to culture the proteins for 1 hour. Next, the plates were washed, and 100 μL of diluted standard or serum samples was added to culture the protein for 2 hours, with blank control set up using the aforementioned method. After the plates were washed, TSLP antibody or COL2AL antibody was added to each well to culture the proteins for 1 hour. At this point in the operation, the plates were washed again, and 100 μL of the substrate solution was added in each well to react with the proteins for 15 minutes at room temperature. The reaction was terminated with the addition of 50 μL sulfuric acid (2 M). The optical density (OD) value at 450 nm of each well were read and recorded using a microplate reader, and TSLP AND COL2AL concentration were calculated based on standard curves.

### 2.9 MTT assay

A MTT assay was applied to detect the cell activity and after the cells had proliferated and fused a Hams F12 was used for diluting the concentration of nucleus pulposus cells to be 1 × 10^5^/mL. The cells were inoculated in 12-well plates (0.2 mL/well), and cultured continuously in a humidified incubator (37 °C, 5% CO_2_). The cells were placed over 96-well plates and then counted based on the fact that each 200 μL contained 200 cells. For the cells in each group a total of 4 96-well plates and 8 duplicate wells were prepared; there were also 4 time intervals points, namely 0, 24, 48, and 72 hours. Once the nucleus pulposus cells in each well adhered to the cell wall, the culture medium was suctioned out and cells were added with 20 μL of MTT (5 g/L). After culturing the cells for another 4 minutes, dimethylsulfoxide (DMSO) (150 μL/well) was added and fully mixed with the cells. Followed by testing the OD value at 570 nm using an enzyme-linked immunosorbent assay 10 minutes later. The result was recorded with time as the abscissa and OD value as ordinate, on the basis of which a precise cell growth curve was drawn.

### 2.10 Antonopulos method

One milliter of nucleus pulposus cells supernatant from the digestive tract was collected after cell proliferation and fusion, and was mixed with 0.5 mL of hydrochloric acid (4 mol/L) and 0.5 mL of protection solution. The cells were underwent a warm bath and hydrolysis at 110 °C for 6 hours, which was followed by the addition of 10 mL of distilled water (pH 6.0) for filtration. The 0.2 mL of filtered solution was collected and 0.4 mL of distilled water, 0.4 mL of sodium carbonate (4 mol/L), and 0.5 mL of 2% acetylacetonate were added to the solution. After mixing the solution, it was boiled for 20 minutes. Once the mixture cooled down 0.5 mL of Her reagent was introduced. The OD value was testified at 530 nm using a spectrophotometer (Type: 721).

### 2.11 Flow cytometry

After inducing apoptosis in cells of each group 10 ng/mL IL-1β for 24 hours, the cells were collected and washed by PBS 3 times. 1 × 10^4^ nucleus pulposus cells were resuspended in binding buffer. The cells were then labeled using Annexin V-PE and 7-AAD (Shanghai Bioon Biotechnology Co., Ltd., Shanghai), they were detected using a FACS Calibur flow cytometer. Cells in the Annexin V+/7-AAD− quadrant were considered as early apoptotic cells, while the cells in the Annexin V+/7-AAD+ quadrant were considered as late apoptotic cells. Both the early and late apoptotic cells were counted together.

### 2.12 Statistical analysis

Clinical data obtained from all patients using a follow-up were encoded onto Microsoft excel (version: 2010) to establish a database. SPSS 20.0 software (SPSS Inc., Chicago, IL) was applied for statistical analysis. Measurement data were represented in terms of standard and deviation and highlighted using a *t* test. The rank-sum test was used for ranked data analysis. The counting data were represented as percentage or rate and compared using a *χ*^2^ test. *P* < .05 was considered to be statistically significant.

## 3 Results

### 3.1 TSLP expression between the case and control groups

**Figure 1:**
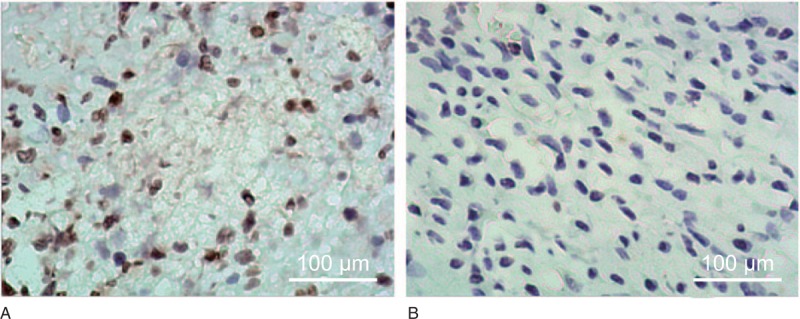
TSLP expression between the case and control groups detected by immunohistochemistry. Note: A, TSLP-positive expression in the case group (×100); B, TSLP-positive expression in the control group (×100); there were 77 cases in the case group and 21 cases in the control group. TSLP = thymic stromal lymphopoietin.

The immunohistochemistry results showed that TSLP was positively expressed in the case group (Fig. 1A) while it was negatively expressed in the control group (Fig. 1B). The TSLP-positive expression rate of the case group was 61.04% (47/77), which was significantly higher than that of the control group (33.33%, 7/21). There was a significant difference between the 2 groups (*χ*^2^ = 5.119, *P* < .001).

### 3.2 Correlation of TSLP-positive expression rate with Pfirrmann grades, Spengler type, and VAS score in LDD patients

The TSLP-positive expression rate of patients with Pfirrmann I, II, III, and IV were 14.29%, 35%, 80.95%, and 95.45%, respectively. The TSLP-positive expression rate increased with an upgrade in the Pfirrmann grade (*χ*^2^ = 8.971, *P* = .030). The TSLP-positive expression rate of patients with lumbar disc herniation, lumbar spondylolisthesis, and lumbar spinal stenosis were 56%, 62.07%, and 65.22%, respectively. However, no correlation was established between the TSLP-positive rate and the degeneration type. In terms of VAS scores, patients were divided into mild (n = 8), moderate (n = 23), and severe (n = 46), and the TSLP-positive expression rates were 12.50%, 30.43%, and 84.78%, respectively. The TSLP-positive expression rate elevated with an increase in the VAS score (*χ*^2^ = 7.750, *P* = .021) (Table 2).

**Table 2:**
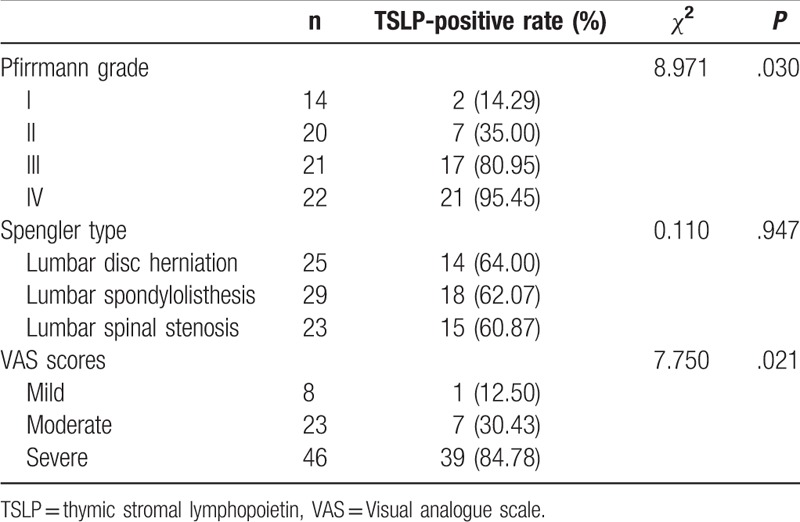
Correlations of TSLP expression with Pfirrmann grades, Spengler type, and VAS scores in LDD patients.

### 3.3 TSLP and TSLPR protein expression in nucleus pulposus tissues between the case and control groups

**Figure 2:**
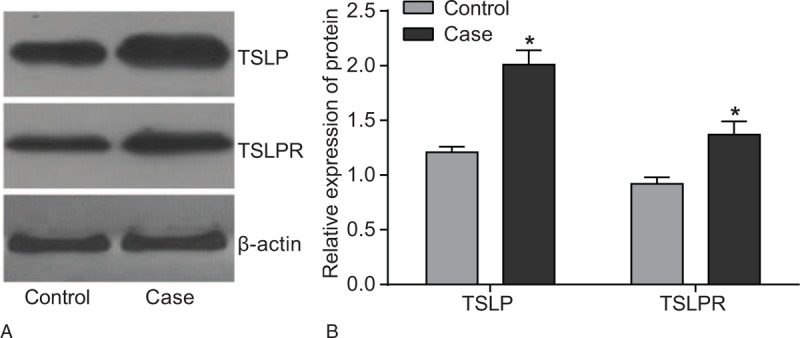
TSLP and TSLPR protein expression in nucleus pulposus tissues between the case and control groups detected by western blotting. Note: ∗, compared with the case group, *P* < .05. TSLP = thymic stromal lymphopoietin, TSLPR = thymic stromal lymphopoietin receptor.

As shown in Fig. 2, according to the results of western blotting, compared with the control group, patients in the case group showed a significantly high TSLP and TSLPR protein expression (both *P* < .05).

### 3.4 SiRNA screening for specific RNA interference

**Figure 3:**
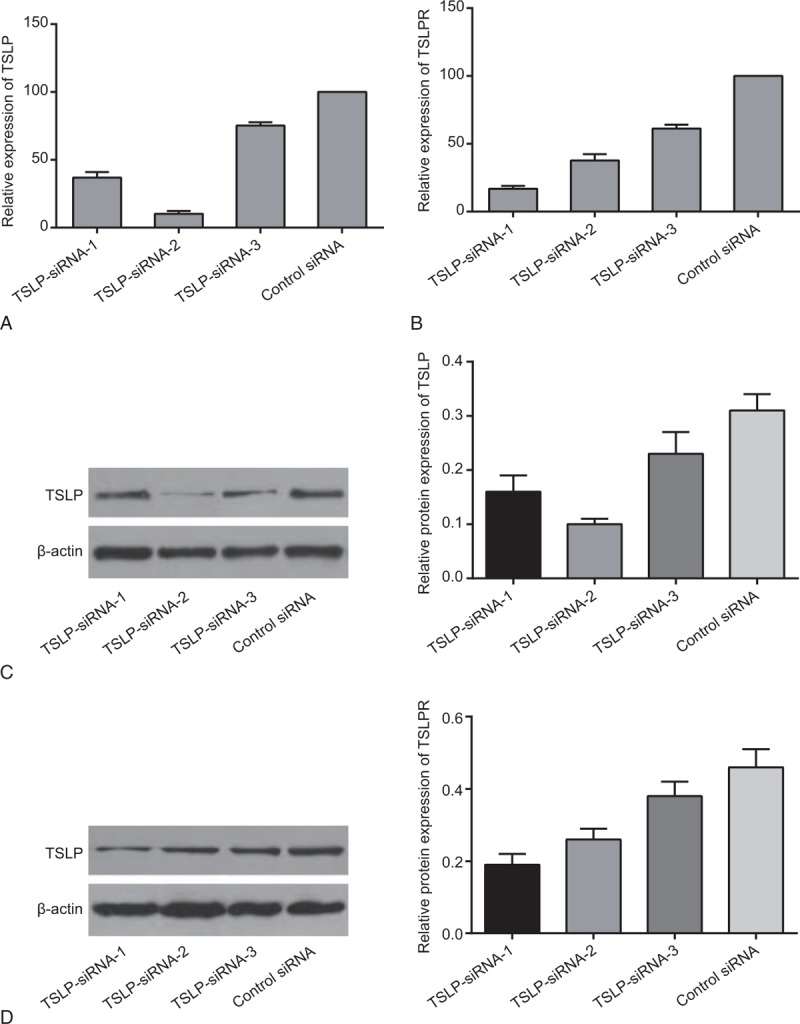
Expression of TSLP in nucleus pulposus cells transfected with siRNA-1 against TSLP, siRNA-2 against TSLP, or siRNA-3 against TSLP, and expression of TSLPR in nucleus pulposus cells transfected with siRNA-1 against TSLPR, siRNA-2 against TSLPR, or siRNA-3 against TSLPR, detected by qRT-PCR and western blotting. Note: A, the mRNA level of TSLP; B, the mRNA level of TSLPR; C, grey value of TSLP protein band and the protein level of TSLP; D, grey value of TSLPR protein band and the protein level of TSLPR; qRT-PCR = quantitative real-time polymerase chain reaction, TSLP = thymic stromal lymphopoietin, TSLPR = thymic stromal lymphopoietin receptor.

2^−ΔΔCt^ methods were used to calculate and analyze the relative expressions of *TSLP* and *TSLPR* gene, and the inhibitory effect of 3 different TSLP siRNAs and 3 different TSLPR siRNAs. Compared with the control siRNA group, the TSLP mRNA-inhibition rate of TSLP siRNA-1 was 63%, the TSLP mRNA-inhibition rate of TSLP siRNA-2 was 90%, and the TSLP mRNA-inhibition rate of TSLP siRNA-3 was 25% (Fig. 3A). In comparison with the siRNA control group, the TSLPR mRNA-inhibition rate of TSLPR siRNA-1 was 83%, the TSLPR mRNA-inhibition rate of TSLPR siRNA-2 was 60%. The TSLPR mRNA-inhibition rate of TSLPR siRNA-3 was 39% (Fig. 3B). The aforementioned results specified that, under 50 nmol/L transfection concentration, TSLP siRNA-2 could effectively inhibit the expression of TSLP mRNA, and TSLPR siRNA-1 could remarkably inhibit the expression of TSLPR mRNA. After TSLP and TSLPR protein expressions were calculated and analyzed using western blotting, it is found that TSLP siRNA-2 had the most remarkable and significant effect on TSLP inhibition (Fig. 3C), and therefore TSLPR siRNA-1 was the best at inhibiting TSLPR (Fig. 3D). Therefore, TSLP siRNA-2 and TSLPR siRNA-1 were selected to inhibit TSLP and TSLPR expression.

### 3.5 TSLP expression among the blank, control, TSLP-siRNA, TSLP-siRNA + TSLPR-siRNA, IgG, and anti-TSLPR groups

**Figure 4:**
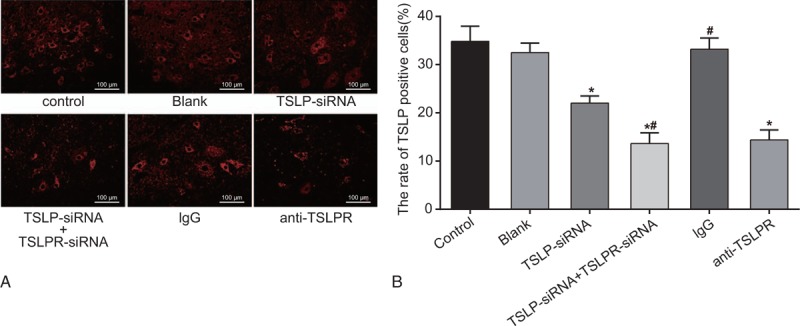
TSLP expression among the blank, control, TSLP-siRNA, TSLP-siRNA + TSLPR-siRNA, IgG, and anti-TSLPR groups. Note: A, TSLP expression in each group detected by immunofluorescence staining; red was TSLP, and blue was DAPI; B, comparison of TSLP-positive expression among each group; ∗, compared with the blank group, *P* < .05; #, compared with the TSLP-siRNA group, *P* < .05;DAPI = 4′,6-diamidino-2-phenylindole, IgG = immunoglobulin G, TSLP = thymic stromal lymphopoietin, TSLPR = thymic stromal lymphopoietin receptor.

According to the result of immunofluorescence, there was no difference in TSLP-positive rate among the blank group, the Control group, and the IgG group, while the TSLP-siRNA group, the TSLP-siRNA + TSLPR-siRNA group, and the anti-TSLPR group showed a lower TSLP-positive rate than the blank group (all *P* < 0.05). Compared to the TSLP-siRNA group, the TSLP-positive rate decreased in the TSLP-siRNA + TSLPR-siRNA group (all *P* < .05). However, there was no difference between the TSLPR-siRNA group and the anti-TSLPR group (Fig. 4).

### 3.6 Serum TSLP level among the blank, control, TSLP-siRNA, TSLP-siRNA + TSLPR-siRNA, IgG, and anti-TSLPR groups

**Figure 5:**
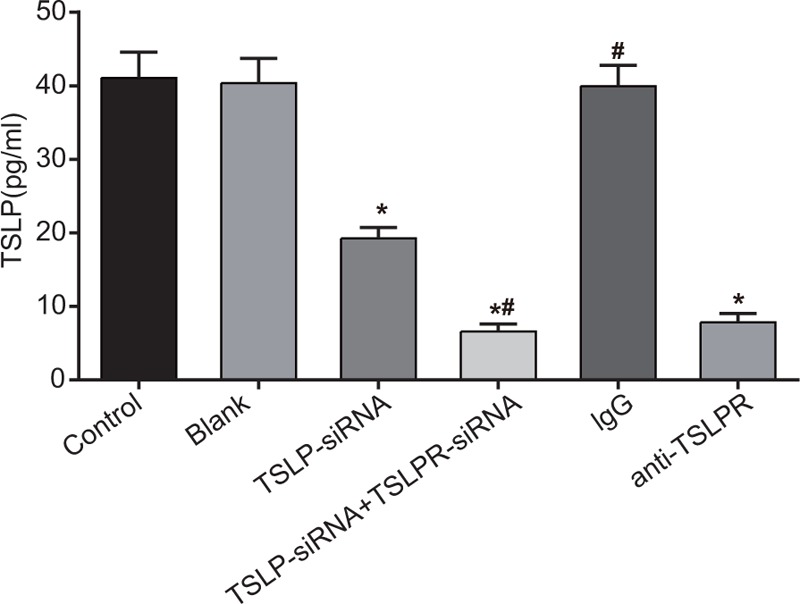
Serum TSLP level among the blank, control, TSLP-siRNA, TSLP-siRNA + TSLPR-siRNA, IgG, and anti-TSLPR groups after cell transfection detected by ELISA. Note: ∗, compared with the blank group, *P* < .05; #, compared with the TSLP-siRNA group, *P* < .05; ELISA = enzyme-linked immunosorbent assay; IgG = immunoglobulin G, TSLP = thymic stromal lymphopoietin; TSLPR = thymic stromal lymphopoietin receptor.

Forty-eight hours after transfecting the nucleus pulposus cells with siRNA no difference was observed in TSLP concentration among the blank group, the control group, and the IgG group, whereas the TSLP-siRNA group, the TSLP-siRNA + TSLPR-siRNA group and the anti-TSLPR group exhibited a drop TSLP concentration than the blank group (*P* < .05). Compared with the TSLP-siRNA group, there was a notable decrease in TSLP concentration level in the TSLP-siRNA + TSLPR-siRNA group (*P* < .05). Besides, the TSLP-siRNA+TSLPR-siRNA group showed the lowest TSLP level among the 6 groups (Fig. 5).

### 3.7 Viability of nucleus pulposus cells among the blank, control, TSLP-siRNA, TSLP-siRNA + TSLPR-siRNA, IgG, and anti-TSLPR groups

A growth curve was drawn using a MTT assay to show the cell viability after cell transfection. The measured OD values of transfected nucleus pulposus cells are shown in Table 3. The results revealed the cell viability of TSLP-siRNA group, the TSLP-siRNA + TSLPR-siRNA group, the control group, the blank group. The IgG group and the anti-TSLPR group were dependent on time at 24, 48, and 72-hour time intervals and the groups had no difference in cell viability at 24 hours (all *P* > .05). The cell viability increased significantly at 48 and 72 hours (all *P* < .05) compared with 24 hours. After cell transfection, the cell viability was higher in the TSLP-siRNA group, the TSLP-siRNA + TSLPR-siRNA group, and the anti-TSLPR group than the control group and the blank group at 48 and 72 hours (all *P* < .05). There were no statistical differences observed among the control group, the bank group, and the IgG group (all *P* > .05). Both the TSLP-siRNA group and the anti-TSLPR group showed poorer cell viability than the TSLP-siRNA + TSLPR-siRNA group (*P* < .05)

**Table 3:**
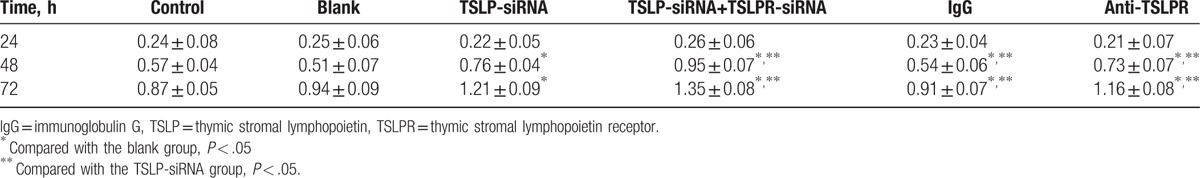
Viability of nucleus pulposus cells among each group detected by MTT assay.

### 3.8 Levels of COL2AL and Aggrecan among the blank, control, TSLP-siRNA, TSLP-siRNA + TSLPR-siRNA, IgG, and anti-TSLPR groups

**Figure 6:**
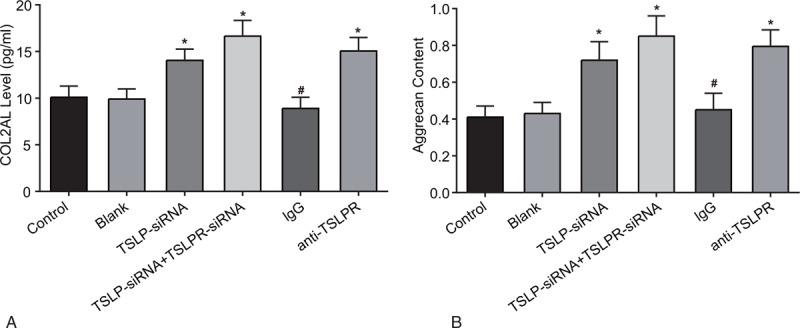
COL2AL and Aggrecan levels in nucleus pulposus cells among the blank, control, TSLP-siRNA, TSLP-siRNA + TSLPR-siRNA, IgG, and anti-TSLPR groups. Note: A, comparison of COL2Al level among each group; B, comparison of Aggrecan level among each group; ∗, compared with the control and blank groups, *P* < .05; #, compared with the TSLP-siRNA group, *P* < .05; COL2AL = type II collagen, IgG = immunoglobulin G, TSLP = thymic stromal lymphopoietin, TSLPR = thymic stromal lymphopoietin receptor.

Compared with the control group and the blank group, there was an increase in COL2AL expression and Aggrecan volume in the TSLP-siRNA group, anti-TSLPR group, and the TSLP-siRNA + TSLPR-siRNA group (all *P* < .05), while no difference was observed between the blank group and the control group. The transfection of TSLP-siRNA and TSLPR-siRNA plasmids could promote nucleus COL2AL expression and aggrecan in pulposus cells (Fig. 6).

### 3.9 Apoptosis of nucleus pulposus cells among the blank, control, TSLP-siRNA, TSLP-siRNA + TSLPR-siRNA, IgG, and anti-TSLPR groups

The results of flow cytometry indicate that there was no difference in the apoptosis rate among the control, blank, and IgG groups.

**Figure 7:**
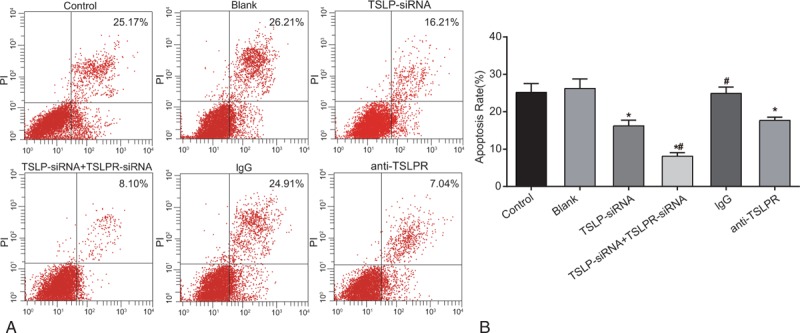
Apoptosis of nucleus pulposus cells among the blank, control, TSLP-siRNA, TSLP-siRNA + TSLPR-siRNA, IgG and anti-TSLPR groups detected by flow cytometry. Note: IgG = immunoglobulin G, TSLP = thymic stromal lymphopoietin, TSLPR = thymic stromal lymphopoietin receptor.

The TSLP-siRNA group, TSLP-siRNA + TSLPR-siRNA group, and anti-TSLPR group showed lower rates of apoptosis compared with the blank group (all *P* < .05). The TSLP-siRNA + TSLPR-siRNA group had lower apoptosis rates compared with the TSLP-siRNA group and the anti-TSLPR group (both *P* < .05). The results indicate that TSLP siRNA, TSLPR-siRNA, and anti-TSLPR could inhibit apoptosis of nucleus pulposus cells (Fig. 7).

## 4 Discussion

LDD leads to high health care costs and has serious implications on general human health.^[18]^ Although the combination of surgery and non-surgical treatment can help in alleviating the symptoms of LDD, it is difficult to achieve satisfactory results due to the high risk. Additionally, the advancements in the field of molecular biology and techniques have led to discovery of many new therapies, such as gene therapy, which has been widely applied to LDD clinical treatment.^[19,20]^ The study found that compared with the control group, TSLP-positive expression was lower than that in the case group. The case group showed a higher TSLPR protein expression. The intervertebral disc, the largest avascular structure surrounded by tissue annulus and isolated by autologous blood circulation, possesses the competence of antigenicity, suggesting a relationship between LDD and autoimmune response.^[21]^ TSLP, an IL-7 cytokine, could be expressed in the epithelial cells present in the Hassall's corpuscles of thymic medulla.^[8]^ It is reported that TSLP can activate dendritic cells (DC), which is the antigen-presenting cells (APC) maintain the immunoregulatory functioning in the induction of Th2 cell polarization and excitation hypersensitivity inflammation.^[22]^ A possible trajectory of the mechanism could be that TSLP induces the expression of INF super-family protein OX40L in DC cells; DC cells promote the generation of Th2 cells in the absence of IL-12 through the interaction between DC and T cell surface receptors OX40L.^[23,24]^ Furthermore, previous study has revealed that TSLP-DC could promote the polarization of Th2 central memory cells at the local inflammation spot and the expression of prostaglandin D2 receptors and other active substances, and therefore could accelerate the process of allergy inflammatory participation.^[25]^ A previous study found that increased level of TSLP could be induced by human epithelial cells through costimulation of inflammatory cytokines, such as TNF-α, IL-1β, and Th2 cytokines.^[26]^ TSLPR is a novel receptor subunit related to sequence to IL-2 receptor common gamma chain.^[27]^ TSLP is capable of binding to a heterodimer receptor complex composed of the IL-7 receptor alpha chain.^[28]^ Therefore, TSLP expression is positively proportional to TSLPR.

The present study found that TSLP-positive rate is associated with a higher Pfirrmann grade and higher VAS score. Pfirrmann disc degeneration grade is a morphologic disc degeneration grading system.^[29]^ The results of the present study were confirmed by Rodrigues et al,^[30]^ who considered discs with a Pfirrmann grade lower than or equal to II to be normal, and those with a Pfirrmann grade higher than II to be degenerated. The VAS score is frequently used in clinical medicine in order to estimate the severity of subjective symptoms.^[31]^ TSLP could result in acceleration of allergy inflammatory; therefore, it is reasonable to assume that a TSLP-positive rate is associated with a higher VAS score.

By the means of this study, it has been highlighted that TSLP-siRNA transfected human nucleus pulposus cells can promote the viability of nucleus pulposus cells and synthesis of COL2AL and Aggrecan. The matrix components are responsible for COL2AL, Aggrecan, and cartilage mechanical properties. Aggrecan, a protective factor in preventing degradation of collagen fibrils and the use of an aggrecanase inhibitor could affect overall cartilage protection.^[32]^ Alteration of matrix components is related to inflammatory cells infiltration and hemorrhage.^[33]^ As a result, lesser inflammation caused by TSLP siRNA can result in an increase of the matrix component, causing increased secretion of Aggrecan and COL2AL. In addition, TSLP-siRNA could inhibit the apoptosis and induction of nucleus pulposus cells in order to reduce the apoptosis rate. The main components of the extracellular matrix disc include collagen with strong toughness (whereas type II collagen covers a larger proportion), proteoglycans, elastin, and water.^[34]^ The occurrence of LDD results in unclear boundaries between annulus and nucleus, reductions in the synthesis of nucleus proteins and carbohydrates, changes in the collagen arrangement and type, and an obvious decrease of the corresponding biological role.^[35]^ It has been demonstrated that IL-1β and TNF-α could induce the apoptosis of nucleus pulposus cells, as well as promote the expression of TSLP.^[22,36]^ RNA interference (RNAi) is a widely used technique that specifically inhibits the expression of the corresponding gene sequences.^[37]^ Thus, apoptosis of nucleus induced by IL-1β was decreased after the inhibition of TSLP activity. Similarly, we observed that anti-TSLPR could promote cell viability, increase COL2AL and Aggrecan levels, and prohibit cell apoptosis. Studies have shown that upregulation of eosinophil surface expression of TSLPR significantly enhanced sensitivity of eosinophils in order to TSLP-mediated degranulation.^[38]^ TSLP signaling was blocked by intratracheal administration of anti-TSLPR antibody before sensitization.^[39]^ Hence, anti-TSLPR coupled with blocked TSLP signaling, had the similar function as TSLP-siRNA.

## 5 Conclusion

In summary, the TSLP positive expression rates of LDD patients in the case group were significantly higher compared with the control group. The TSLP expression indirectly reflects the degree of degeneration for histological level in intervertebral disc. The transfection of TSLP-siRNA or anti-TSLPR significantly inhibits the apoptosis and induction of nucleus pulposus cells, could potentially serve as a new target for LDD treatment. TSLP is a growth factor secreted outside the cell; however, the study fails to detect TSLP levels in blood samples. Consequently, the TSLP level determined in the study could not be completely accurate in comparison. Hence, further analysis and investigation are required to confirm the results our study.
